# Heart rate reduction during voluntary deep diving in free-ranging loggerhead sea turtles

**DOI:** 10.1242/jeb.246334

**Published:** 2024-03-07

**Authors:** Ayaka Saito, Chihiro Kinoshita, Kino Sakai, Katsufumi Sato, Kentaro Q. Sakamoto

**Affiliations:** Atmosphere and Ocean Research Institute, The University of Tokyo, 5-1-5 Kashiwanoha, Kashiwa, Chiba 277-8564, Japan

**Keywords:** Diving physiology, Diving bradycardia, Electrocardiogram, *Caretta caretta*, Free-ranging

## Abstract

Air-breathing vertebrates exhibit cardiovascular responses to diving including heart rate reduction (diving bradycardia). Field studies on aquatic mammals and birds have shown that the intensity of bradycardia can vary depending on diving behaviour, such as the depth of dives and dive duration. However, in aquatic reptiles, the variation in heart rate during deep dives under natural conditions has not been fully investigated. In this study, we released five loggerhead sea turtles (*Caretta caretta*) outfitted with recorders into the sea and recorded their electrocardiogram, depth, water temperature and longitudinal acceleration. After 3 days, the recorders automatically detached from the turtles. The heart rate signals were detected from the electrodes placed on the surface of the plastron. The mean (±s.d.) heart rate of 12.8±4.1 beats min^–1^ during dives was significantly lower than that of 20.9±4.1 beats min^–1^ during surface periods. Heart rate during dives varied with dive depth, although it remained lower than that at the surface. When the turtle dived deeper than 140 m, despite the relatively high flipper stroke rate (approximately 19 strokes min^–1^), the heart rate dropped rapidly to approximately 2 beats min^–1^ temporarily. The minimum instantaneous heart rate during dives was lower at deeper dive depths. Our results indicate that loggerhead sea turtles show variations in the intensity of diving bradycardia depending on their diving behaviour, similar to that shown by marine mammals and birds.

## Teaser

**Summary:** The heart rate reduction in free-ranging loggerhead turtles during diving is affected by dive depth.

## INTRODUCTION

Cardiovascular regulation is important in aquatic air-breathing vertebrates during diving. In aquatic mammals and birds, the heart rate drops upon diving via the parasympathetic nervous system, a phenomenon called diving bradycardia (Butler and Jones, 1997; Ponganis, 2015). During forced submersion, diving bradycardia can be severe, allowing aquatic mammals and birds to selectively distribute oxygen to the oxygen-sensitive organs and tissues (Butler and Jones, 1997; Ponganis, 2015). Although diving bradycardia is moderate during voluntary diving compared with that during forced submersion, field studies in marine mammals and birds have shown that the intensity of diving bradycardia varies among species and according to their diving behaviour, including the depth of dives, dive duration and underwater exercise. For example, the heart rate is further reduced during deeper and longer dives (Froget et al., 2004; McDonald and Ponganis, 2014; Wright et al., 2014). Furthermore, exercise is associated with an increase in heart rate, whereas the degree of bradycardia varies with the depth at which exercise takes place, suggesting that heart rate is influenced by multiple factors, including dive depth, dive duration and exercise, rather than solely the nature of submersion (Williams et al., 2015). This cardiovascular regulation allows marine mammals and birds to use their limited oxygen stores efficiently and dive deep and long (Williams et al., 2015; Davis and Williams, 2012).

However, the variation in heart rate of aquatic reptiles during diving is not fully understood. Severe bradycardia occurred during prolonged dives in experiments that examined forced submersion (2–4 beats min^–1^ in freshwater turtles; White and Ross, 1966; 1 beat min^–1^ in green sea turtles; Berkson, 1966) and captive voluntary dive (2.3 beats min^–1^ in freshwater turtles; Belkin, 1964). Within the range of routine dives, such further heart rate reduction is considered unnecessary in captive freshwater turtles (Belkin, 1964) and loggerhead sea turtles (Williams et al., 2019) because their heart rate did not correlate with dive duration. However, specifically in deep dives under natural conditions, the variation of heart rates in free-ranging aquatic reptiles has not been examined. Although field studies on aquatic reptiles have shown a slight reduction in heart rate during diving, these studies were conducted on species that inhabit shallow water, such as freshwater crocodiles (Seebacher et al., 2005), American alligators (Smith et al., 1974) and sea snakes (Heatwole et al., 1979), or on leatherback sea turtles during the interesting interval during which they dived to relatively shallower levels than the levels to which they dive normally during foraging periods (Southwood et al., 1999). Among marine reptiles, sea turtles have outstanding diving abilities. For example, loggerhead sea turtles have been reported to dive for as long as 10 h at one time (Broderick et al., 2007) and to dive to depths greater than 340 m (Narazaki et al., 2015). In addition, they perform V- and U-shaped dives, which are common with penguins and seals. Loggerhead turtles conduct several other types of dives, such as those in which they descend to a maximum dive depth and then gradually ascend, or ascend to a certain depth and stay around that depth (Minamikawa et al., 1997). Furthermore, when loggerhead turtles submerged in a water tank, their heart rates are reduced via the parasympathetic nervous system, similar to aquatic mammals and birds (Saito et al., 2022). Therefore, characteristic changes in the heart rate may occur when loggerhead turtles dive under natural conditions at sea.

In this study, for the first time, we recorded the electrocardiograms of loggerhead turtles in the sea and investigated the changes in heart rate when they performed deep and long dives. The heart rates and diving behaviours of five juvenile turtles in the Sanriku coastal area of the western North Pacific were measured using a non-invasive method of measuring electrocardiograms (Sakamoto et al., 2021; Kinoshita et al., 2022) and an automated tag release system (Narazaki et al., 2009; Watanabe et al., 2004). We examined the hypotheses that heart rates are lower during diving than when at the surface for breathing, and that heart rates are further reduced during deeper and longer dives. In addition, we analysed the detailed profile of heart rate according to dive depth.

## MATERIALS AND METHODS

### Animals

Five juvenile loggerhead turtles, *Caretta caretta* (Linnaeus 1758), were used in this study. The mean±s.d. body mass of the five turtles was 59.7±10.9 kg (46.0–76.3 kg), and the sexes were not determined (Table 1). These turtles were incidentally captured by commercial set nets in the Sanriku coastal area of Japan during the summers of 2021 and 2022. The study site was a summer-restricted foraging ground for loggerhead turtles (Narazaki et al., 2015). Once safely rescued, the turtles were promptly transferred to the marine station of the University of Tokyo (Otsuchi Coastal Research Center, Atmosphere and Ocean Research Institute, The University of Tokyo; 39°21′05″ N, 141°56′04″ E). This study was conducted as part of a ‘tag and release’ programme, in which loggerhead turtles caught by set nets through bycatch in the Sanriku Coast were handed over to researchers by fishers. After the turtles were tagged with metal ID labels, they were kept individually in outdoor water tanks (1×2×1 m) for a maximum of 1–2 months. The turtles showed no external injuries and no unusual behaviours during the captive period. All experimental procedures were approved by the Animal Ethics Committee of the Atmosphere and Ocean Research Institute at the University of Tokyo (permission nos P21-13 and P22-12).

**Table 1.**
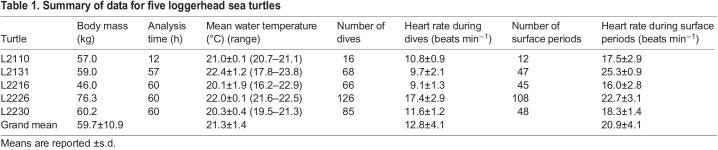
Summary of data for five loggerhead sea turtles

### Instruments and fieldwork procedure

An electrocardiogram (ECG) was recorded at 250 Hz using an ECG logger (ECG400-DT; Little Leonardo, Tokyo, Japan; 21×64×23 mm, width×length×height, 60 g mass in air; and ECG400-D3GT; Little Leonardo; 31×67×17 mm, width×length×height, 61 g mass in air). In addition, the longitudinal acceleration was recorded at 16 Hz, temperature at 1 Hz and depth at 1 Hz using an accelerometer (W1000-3MPD3GT; Little Leonardo; 26×175 mm, diameter×length, 140 g in air). The ECG signals of the turtles were recorded using a previously described non-invasive method of attaching two electrode patches to the plastron of the turtles (Sakamoto et al., 2021; Kinoshita et al., 2022). The lead wires attached to the electrodes were arranged from the plastron along the side of the body to the carapace. These wires were connected to lead wires extending from the ECG logger across a time-scheduled release mechanism (BCR-300; Little Leonardo). The ECG logger and accelerometer were fixed to a float (100×230 mm, width×length, 210 g in air) along with a satellite-compatible transmitter (SPOT6; Wildlife Computer; 20×56 mm, diameter×length, with an 18 cm semi-rigid wire antenna, 41 g in air) and a VHF radio transmitter (Advanced Telemetry System; 12×55 mm, diameter×length, with a 35 cm semi-rigid wire antenna, 27 g in air). The logger package was attached to a plastic mesh along the longitudinal axis of the carapaces of the turtles using a plastic cable tie. The cable tie was connected to a time-scheduled release mechanism (RC-300-150; Little Leonardo). After attaching instruments, the turtles were released from Heita Bay (39°14′50.1″ N 141°53′26.4″ E). Time-scheduled release mechanisms were programmed to be activated 72 h after deployment. At the activation time, an electric charge incised the plastic cable, releasing the logger package from the turtles (Narazaki et al., 2009; Watanabe et al., 2004) and disconnecting the lead wires between the electrodes and the ECG logger. The logger package was located using SPOT6 and VHF radio signals.

### Analysis

ECG, depth, temperature and longitudinal acceleration were analysed using IGOR Pro version 8.04 (Wavemetrics, Portland, OR, USA) with the Ethographer program package (Sakamoto et al., 2009). Data within a 12-h period after release were excluded to eliminate the handling effects of the instrument attachments. In the case of turtle L2110, the data were analysed only from 12 h after release until 08:50 h (for approximately 12 h) because the ECG logger stopped recording during the measurement. In addition, the ECG data of turtle L2131 were lost for approximately 3 h during data processing in the device; therefore, this period was excluded from the analysis.

**Fig. 1.**
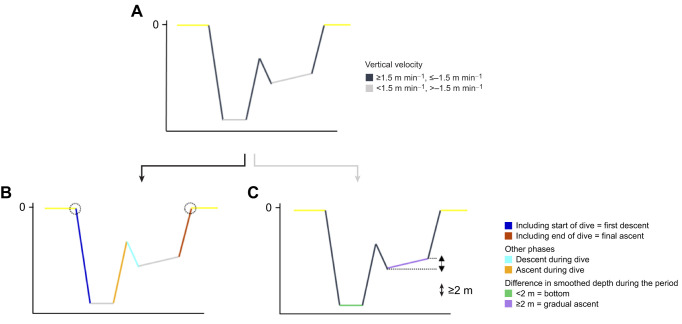
**Typical diagram of the criteria for dividing a dive into segments.** (A) Vertical velocity. Black and grey lines indicate a period of vertical velocity 1.5 m min^−1^ or above and −1.5 m min^−1^ or below (the descent and ascent phases), and between 1.5 and −1.5 m min^−1^, respectively. (B) The period of the descent and ascent phases is then divided by whether it includes the start or the end of the dive (dashed circle). (C) In addition, a period of the vertical velocity between 1.5 and −1.5 m min^−1^ was divided based on the threshold of a difference in smoothed depth (2 m). See Analysis in Materials and Methods for definitions of segments. Yellow line indicates surface periods and other lines indicate each segment.

Dives were considered to be at a depth of 1 m or greater. We analysed dives lasting 5 min or more because the heart rate tends to change gradually over several minutes. Surface periods were defined as the periods when the turtles were at the surface between dives for 1 min or more. Of these, 97% were less than 10 min; therefore, surface periods longer than 10 min were excluded from the analysis to avoid the possibility that the turtles may have conducted shallower dives than the 1-m depth threshold or basked at the surface. The maximum dive depth was used as the representative value of depth during dives. For a detailed analysis of the changes in heart rate during dives, the dives were divided into six segments: (1) first descent, (2) final ascent, (3) descent during dive, (4) ascent during dive, (5) bottom and (6) gradual ascent, during which the sea turtles descended to the maximum dive depth and then gradually ascended. To determine these segments, depth data were first smoothed at 60-s intervals, and the difference in smoothed depth per minute was calculated as the vertical velocity. Subsequently, the phases with vertical velocity 1.5 m min^−1^ or above and −1.5 m min^−1^ or below were regarded as the descent and ascent phases, respectively (Fig. 1A). The period of the descent phase including the start of the dive was defined as first descent, and the period of the ascent phase including the end of the dive was defined as final ascent (Fig. 1B). Other descent and ascent phases were defined as descent and ascent during dive, respectively (Fig. 1B). Moreover, the period with the vertical velocity between 1.5 and −1.5 m min^−1^ was considered as bottom (Fig. 1C). Among these periods, a difference in smoothed depth of 2 m or more during the period was considered as gradual ascent (Fig. 1C). Finally, the dive periods, surface periods and six segments were checked manually.

Longitudinal acceleration data were filtered to extract longitudinal high-frequency components (mainly caused by dynamic movements) as information on the flipper stroking activity (Sato et al., 2011). The filter was defined using the power spectral density and a continuous wavelet transform filter. The flipper stroke rate was calculated as the number of strokes per minute.

The ECGtoHR program package (Sakamoto et al., 2021) was used to process ECG data. Noise was removed from the ECG data using a bandpass filter, and R waves were detected. The instantaneous heart rates in beats min^−1^ were calculated as the reciprocal of the time interval between two sequential R-waves (RR interval). Heart rate per minute was calculated as the median value of the instantaneous heart rates per minute. For each turtle, the heart rate during each period (dives, surface periods and six segments) was calculated by dividing the total number of heartbeats per period by the duration of each period. Mean values were calculated for each turtle across all recorded dives, and these means were further averaged to obtain a grand mean. For data analysis, linear mixed-effects models including individual turtles as a random effect were used, and the *P*-values were calculated using Satterwaite's method. We compared the mean heart rates during dive and surface periods. We also examined the relationships between the minimum instantaneous heart rate during dives and the parameters of diving behaviour (the maximum dive depth, dive duration and the flipper stroke rate during dives) in all turtles across all recorded dives. As the minimum instantaneous heart rate decreased exponentially with increasing maximum diving depth, the maximum dive depth was transformed using a common logarithm and used for the analysis. The mean water temperature during dives and body mass of turtles were also included as fixed effects. The models were compared by maximum likelihood using Akaike's information criterion (AIC). The model without the mean water temperature during dives and body mass had the lowest AIC. Therefore, we adopted a model that included the three variables of maximum dive depth, dive duration and the flipper stroke rate to explain the minimum instantaneous heart rate during dives. Differences in heart rate between the six segments (first descent, final ascent, descent during dive, ascent during dive, bottom and gradual ascent) were examined using a linear mixed-effects model, followed by the Tukey–Kramer *post hoc* test. Differences were considered statistically significant at *P*<0.05. The values are expressed as means±s.d. Statistical analysis was performed using packages lme4 (Bates et al., 2015) and multcomp (Hothorn et al., 2008) in R version 4.0.0 (https://www.r-project.org/).

## RESULTS

**Fig. 2.**
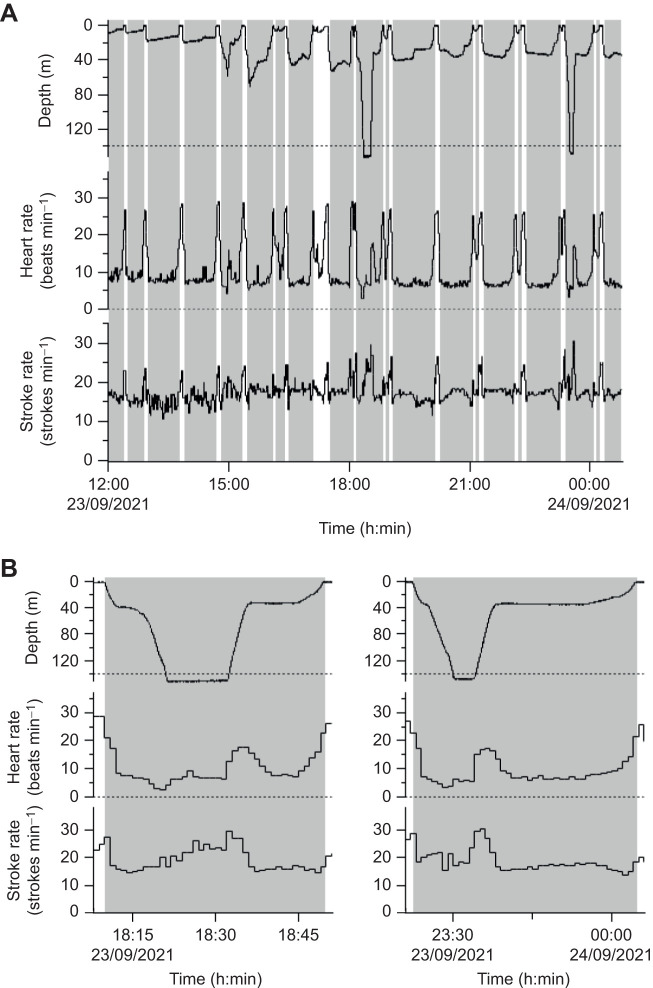
**Depth, heart rate and flipper stroke rate profiles of loggerhead sea turtle L2131.** Profiles for (A) approximately 48 h after release and (B) two dives deeper than 140 m. Shaded areas indicate the dive periods. The dashed line of the depth profiles indicates a 140 m line and that of the heart rate profile indicates a zero line.

**Fig. 3.**
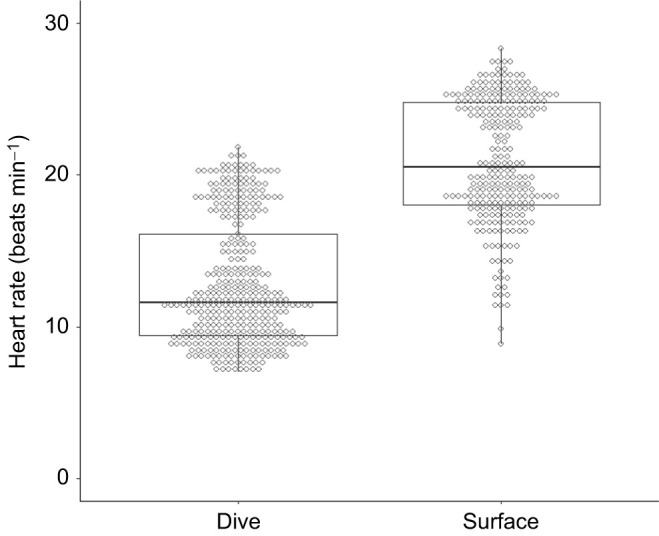
**Mean (+s.d.) heart rate during dives (*n*=361) and during surface periods (*n*=260) for five loggerhead sea turtles.** The top and bottom of each box indicate quartiles, and the lines indicate median values. The whiskers extending from the top and bottom of each box mark the 1.5-fold interquartile range.

**Fig. 4.**
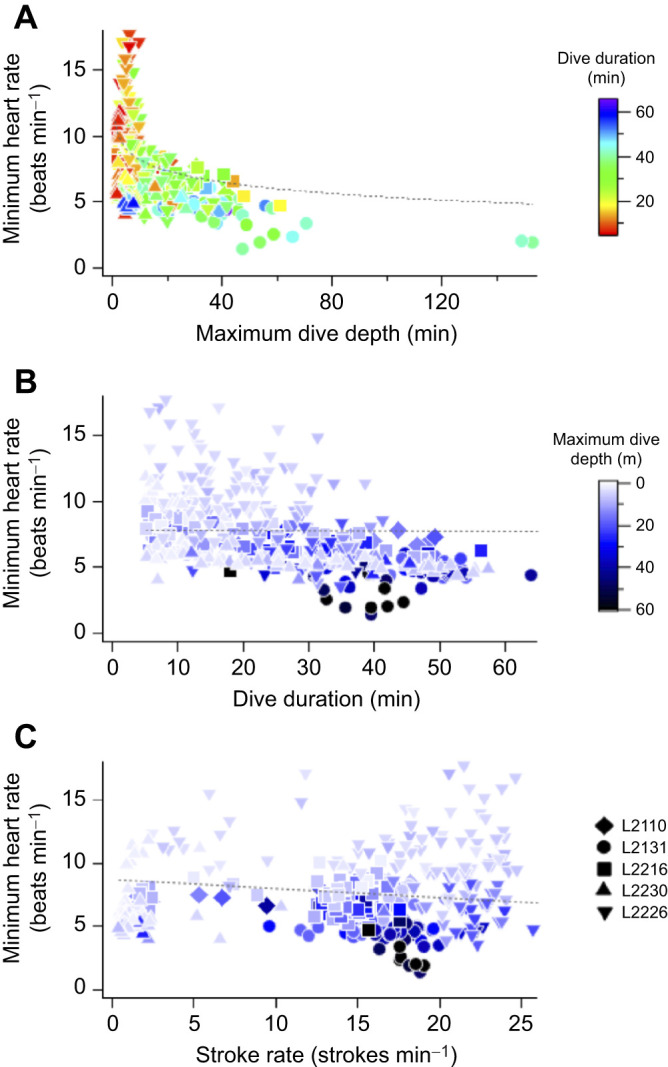
**The minimum instantaneous heart rate during dives versus maximum dive depth, dive duration and flipper stroke rate for five loggerhead sea turtles (*n*=361 dives).** (A) Maximum dive depth, (B) dive duration and (C) flipper stroke rate. Colour scales represent dive duration in A, and the maximum dive depth in B and C. Dashed lines were calculated by linear mixed-effects model (other variables were calculated using mean values).

Five turtles conducted 361 dives in total for an analysis time of 249 h (Table 1). Dive durations ranged from 5.1 to 63.9 min for all individuals. The dive depths occasionally exceeded 140 m in turtle L2131 and ranged from 1.8 to 153 m in all individuals. Dive duration and depth were within the range of loggerhead turtles in the western North Pacific during summer (Narazaki et al., 2015). Regardless of the type of dive, the heart rates of the turtles decreased immediately upon diving (Fig. 2A). The mean heart rate during dives (12.8±4.1 beats min^–1^, *n*=361) was significantly lower (*P*<0.001, *F*_1,616.1_=1062.4; Fig. 3, Table 2) than that during surface periods (20.9±4.1 beats min^–1^, *n*=260). The heart rate was lower by 39% during dives compared with those during surface periods, although the reduction rate varied from 23% to 61% among individuals (Table 1). Generally, the instantaneous heart rates during dives were minimal, either at the maximum dive depth or during the latter half of the dives. The minimum instantaneous heart rate during dives decreased significantly with deeper dive depth (*P*<0.001, *F*_1,359.7_=50.5; Fig. 4A, Table 2). In particular, when the turtle dived deeper than 140 m, despite the relatively high flipper stroke rate during dives (approximately 19 strokes min^–1^), its heart rate dropped rapidly to approximately 2 beats min^–1^ temporarily (Fig. 2B). The minimum instantaneous heart rate during dives was also significantly correlated with dive duration (*P*<0.001, *F*_1,358.7_*=*22.8; Fig. 4B, Table 2) and flipper stroke rate (*P*<0.01, *F*_1,355.6_*=*7.5; Fig. 4C, Table 2). The model showed that for the range of diving behaviours in the present study (other variables were calculated using mean values), the minimum instantaneous heart rate was 53% lower at the maximum dive depth (153 m) than at the minimum dive depth (1.8 m). Meanwhile, the model showed that the minimum instantaneous heart rate was lower by less than 1% owing to increasing dive duration (5.1–63.9 min) and by 20% owing to increasing flipper stroke rate (0.4–25.6 strokes min^−1^).

**Table 2.**
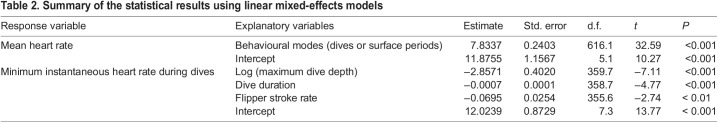
Summary of the statistical results using linear mixed-effects models

**Fig. 5.**
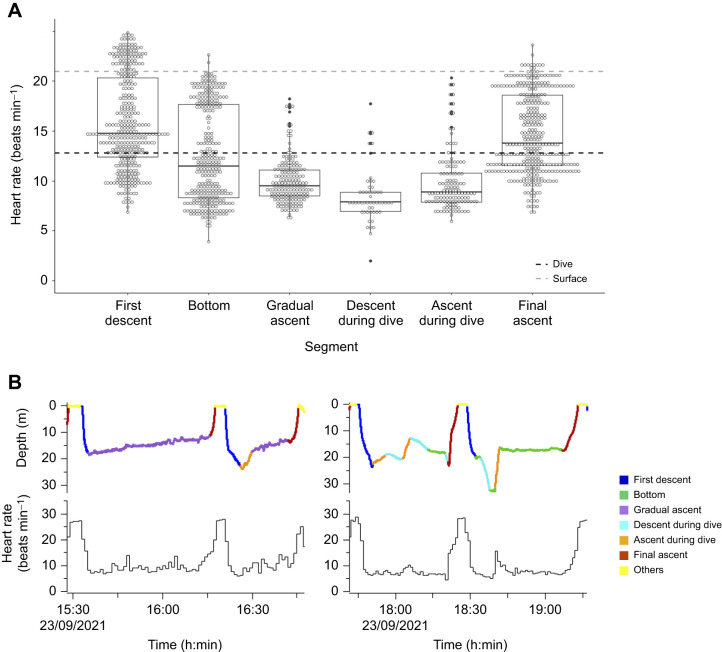
**Comparison of heart rate during six dive segments.** (A) Comparison of heart rate during first descent (*n*=361), bottom (*n*=300), gradual ascent (*n*=175), descent during dive (*n*=52), ascent during dive (*n*=135) and final ascent (*n*=361) for five loggerhead sea turtles. The top and bottom of each box indicate quartiles, and the lines indicate median values. The whiskers extending from the top and bottom of each box mark the 1.5-fold interquartile range. Black and grey dashed lines indicate the mean heart rate during dives and during surface periods, respectively. (B) Example of depth and heart rate profiles with divided segments (turtle L2131). Colours indicate each segment (see Analysis section in Materials and Methods for definitions of segments) and ‘others’, which includes surface periods and periods excluded from the analysis.

Although the heart rate dropped rapidly at the onset of diving and remained lower than that at the surface, it varied with depth during dives (Fig. 2). For example, when the turtles descended to a maximum dive depth and then briefly ascended to a certain depth, the heart rate increased; however, following the bottom phase, the heart rate decreased again and then remained around that value (Figs 2B and 5B; at approximately 18:30–). Further analysis of the heart rate profile according to changes in depth showed that the heart rate differed among the six segments (*P*<0.01, *F*_5,1379.1_=142.6; Fig. 5). During dives, the heart rate was highest at the onset of dives on descent and at the end of dives on ascent. The mean heart rates during both the first descent (15.9±4.7 beats min^–1^, *n*=361) and final ascent (14.7±4.0 beats min^–1^, *n*=361) were significantly different from those at other periods (*P*<0.001). Even during the first descent and final ascent, the mean heart rates were 24 and 30%, respectively, which were lower than those during surface periods. Meanwhile, the bottom and gradual ascent phases dominated dive duration, and the mean heart rates during bottom (12.5±4.7 beats min^–1^, *n*=300) and gradual ascent (10.0±2.3 beats min^–1^, *n*=175) were 40% and 52%, respectively, lower than that during surface periods. In addition, the mean heart rate during bottom was significantly higher than that during gradual ascent (*P*<0.001) and descent during dives (8.5±2.7 beats min^–1^, *n*=52; *P*<0.001) but not ascent during dives (9.9±3.0 beats min^–1^, *n*=135; *P*=0.64). The mean heart rate during gradual ascent was significantly higher than that of ascent during dives (*P*<0.05) but not of descent during dives (*P*=0.86). Significant differences were observed in the mean heart rate of ascent and descent during dives (*P*<0.05).

## DISCUSSION

We obtained the first ECG data of free-ranging hard-shell sea turtles in the sea and investigated the variation in the heart rate of aquatic reptiles during deep and long diving. The heart rate of turtles was significantly lower by 39% during dives compared with during surface periods (Fig. 3, Table 2); however, the heart rate varied within and between individuals. The degree of reduction in heart rate in free-ranging loggerhead sea turtles was greater than that in leatherback sea turtles (30%; Southwood et al., 1999), whereas it was lower than that in California sea lions (52–70%; McDonald and Ponganis, 2014), blue whale (68–84%; Goldbogen et al., 2019), king penguins (46%; Froget et al., 2004) and emperor penguins (68%; Meir et al., 2008). Despite the relatively moderate reduction rate, the heart rate of loggerhead sea turtles, similar to that in aquatic mammals and birds, decreased during dives at sea. Furthermore, the minimum instantaneous heart rate during dives decreased with greater dive depth in loggerhead turtles (Fig. 4A, Table 2), unlike in leatherback turtles, which showed only a weak negative correlation between heart rate and dive depth (Southwood et al., 1999). In aquatic mammals and birds, negative correlations have been found between diving heart rate and dive depth (Williams et al., 2017, 2015). Although comparing the degree of relationships might be challenging because of differences in analysis methods, the minimum instantaneous heart rate abruptly decreased during dives to depths of approximately 20–50 m in narwhales (Williams et al., 2017), bottlenose dolphins and Weddell seals (Williams et al., 2015), which is consistent with the present results for turtles (Fig. 4A). Although heart rate varied among species, the relationship between heart rate and dive depth was similar across species. However, contrary to our hypothesis, dive duration had a relatively small effect on the minimum instantaneous heart rate (Fig. 4B, Table 2). This may be because loggerhead turtles performed various types of dives, resulting in a greater variation in the minimum heart rates depending on the dive depth, even when the turtles dived for a similar time duration. Although sea turtles are ectotherms, the water temperature had no significant effect on the minimum heart rate. This could be attributed to the narrow range of the mean water temperature during the dive (20.1–22.4°C; Table 1), though the turtles experienced temporarily low water temperatures during dive. Similarly, body mass seemed not to affect heart rate owing to the narrow range of body masses in the present study (46.0–76.3 kg; Table 1).

The present results on the flipper stroke rate differed from those of previous studies which indicated that heart rate increased upon the onset of underwater exercise in free-ranging sea snakes (Heatwole et al., 1979) and captive experiments on sea turtles (Williams et al., 2019; Okuyama et al., 2020). In sea turtles, heart rate is thought to elevate upon the onset of underwater exercise and increase linearly with exercise (Okuyama et al., 2020) because their major oxygen stores are their lungs (Lutz and Bentley, 1985) and they need to transport oxygen to muscles from their lungs. The mean heart rate when loggerhead turtles dived at sea was higher than that when they submerged in captivity; the mean heart rates during diving in captivity (body mass, 58.5±18.9 kg; water temperature during dive, 21.4±1.5°C) were 6.4 beats min^–1^ at rest and 10.9 beats min^–1^ when feeding underwater (Williams et al., 2019), whereas the heart rate in the present study was 10.0 beats min^–1^ even during gradual ascent, in which the flipper stroke rate was the lowest among the six segments. Thus, their metabolic load is higher when loggerhead turtles swim under natural conditions, and hence, their basal heart rate may be relatively higher than in captive conditions. However, the further metabolic load did not seem to influence their heart rate while swimming at depth. The minimum instantaneous heart rate was almost independent of the flipper stroke rate (Fig. 4C), and the changes in the flipper stroke and heart rates appeared to be mismatched over time (Fig. 2). When the turtle dived deeper than 140 m, despite the relatively high flipper stroke rate, the heart rate dropped rapidly to approximately 2 beats min^–1^ temporarily (Fig. 2B). Moreover, heart rate during dives remained lower than that during surface periods, regardless of changes in heart rate with dive depth (Fig. 5). Although the metabolic load was expected to increase, specifically during the ascending and descending periods, the heart rate did not markedly increase during either the first descent or final ascent compared with that during surface periods. Because sea turtles have a lower metabolic rate than mammals (Lutcavage et al., 1987), heart rate and flipper stroke rate may not be directly correlated under natural conditions, which constitute a more complex environment than in the water tank experiment. Another possible explanation for these results is that the effect of flipper stroke rate on heart rate decreases with dive depth (Williams et al., 2015). Interestingly, the heart rate increased when the turtles briefly ascended to a certain depth; however, following a gradual ascent or bottom phase, the heart rate decreased again and then remained near that value. Once the heart rate simply changes with dive depth, it should remain around the increased value, instead of increasing and then falling. Therefore, although the basal heart rate under natural conditions was relatively higher compared with that in captive conditions, the heart rate of loggerhead turtles may be regulated by the combined effects of dive depth and activity for deep diving while swimming, similar to aquatic mammals (Williams et al., 2015). As loggerhead turtles perform various types of dives, a more detailed analysis of the variation in heart rate associated with changes in dive depth and activity during dives may reveal the characteristic regulation of heart rate.

Although the heart rate of loggerhead turtles at sea was found to vary according to diving behaviour, a severe reduction in heart rate occurred when the turtles dived greater than 140 m. During prolonged forced diving, heart rate dropped to 1–4 beats min^–1^ in freshwater turtles (White and Ross, 1966) and green sea turtles (Berkson, 1966), and the present results were comparable to these previous results. Such severe bradycardia has also been reported in field studies of marine mammals and birds. Instantaneous heart rate markedly decreased during deeper and longer dives in California sea lions (6–10 beats min^–1^; McDonald and Ponganis, 2014), emperor penguins (8 beats min^–1^; Wright et al., 2014) and grey seals (2 beats min^–1^; Thompson and Fedak, 1993). Previous studies suggest that such bradycardia and the associated reduction in muscle blood flow have a potential advantage for limiting pulmonary gas exchange, conserving lung and blood oxygen and limiting nitrogen absorption, which increases the risk of decompression sickness (McDonald and Ponganis, 2014; Wright et al., 2014). In the present study, almost all dives conducted by loggerhead turtles were aerobic except for three dives above the calculated aerobic dive limit (54–59 min), as estimated from the metabolic rate during diving (for each body mass, at 100% activity ratio; Kinoshita et al., 2018; Lutz and Bentley, 1985). Captive experiments in harbour seals (Elliott et al., 2002) and muskrats (Signore and Jones, 1995) have shown that even when parasympathetic blockers are administered and diving bradycardia is inhibited, animals are still able to make routine aerobic dives, suggesting that bradycardia during routine dives could utilise reserve oxygen stores in case of emergencies, such as unexpected extension diving (Elliott et al., 2002). Even considering that loggerhead turtles have the ability to tolerate hypoxia (Lutz et al., 1980; Lutz and Bentley, 1985), the utilisation of oxygen stores and the risk of decompression sickness in air-breathing vertebrates could be the primary limitations to their diving ability. Therefore, although the findings of the present study alone do not explain the function of severe bradycardia in sea turtles, diving bradycardia can be considered a response to these limitations. However, loggerhead turtles have several different physiological traits from those of aquatic mammals and birds; for example, sea turtles have muscle myoglobin concentrations similar to those of terrestrial animals, and their major oxygen stores are their lungs (Lutz and Bentley, 1985). In addition, anoxia and temperature acclimation may influence heart rate during prolonged anoxia in freshwater turtles (Stecyk and Farrell, 2007). Therefore, it is necessary to further investigate the function of diving bradycardia in sea turtles considering such differences in traits and to confirm the control of diving bradycardia. Further studies on the effects of the inhibition of the cardiovascular response on the heart rate and diving behaviour of sea turtles under natural conditions would help us understand the control of diving bradycardia.

In summary, the present results show that heart rates were lower during dives than when at the surface for breathing, and heart rates were further reduced during deeper dives in free-ranging loggerhead sea turtles under natural conditions. Particularly, when the turtles dived to deep depths, severe bradycardia occurred, similar to that during forced diving. These results suggest that, similar to marine mammals and birds, loggerhead sea turtles exhibit variations in the degree of diving bradycardia depending on their diving behaviour including dive depth. Although the function of diving bradycardia in aquatic reptiles requires further investigation, such regulation of heart rate may be important for diving in aquatic air-breathing vertebrates.
